# P2X7 Receptor and Purinergic Signaling: Orchestrating Mitochondrial Dysfunction in Neurodegenerative Diseases

**DOI:** 10.1523/ENEURO.0092-22.2022

**Published:** 2022-11-10

**Authors:** Alexsandra S. Zelentsova, Alexei V. Deykin, Vladislav O. Soldatov, Anastasia A. Ulezko, Alina Y. Borisova, Veronika S. Belyaeva, Marina Y. Skorkina, Plamena R. Angelova

**Affiliations:** 1 Belgorod State University, Belgorod 308015, Russia; 2 Quine Square Institute of Neurology, London WC1N 3BG, United Kingdom

**Keywords:** α-synuclein, exosomes, mitochondrion, oxidative stress, purinergic metabolome, tau protein

## Abstract

Mitochondrial dysfunction is one of the basic hallmarks of cellular pathology in neurodegenerative diseases. Since the metabolic activity of neurons is highly dependent on energy supply, nerve cells are especially vulnerable to impaired mitochondrial function. Besides providing oxidative phosphorylation, mitochondria are also involved in controlling levels of second messengers such as Ca^2+^ ions and reactive oxygen species (ROS). Interestingly, the critical role of mitochondria as producers of ROS is closely related to P2XR purinergic receptors, the activity of which is modulated by free radicals. Here, we review the relationships between the purinergic signaling system and affected mitochondrial function. Purinergic signaling regulates numerous vital biological processes in the CNS. The two main purines, ATP and adenosine, act as excitatory and inhibitory neurotransmitters, respectively. Current evidence suggests that purinergic signaling best explains how neuronal activity is related to neuronal electrical activity and energy homeostasis, especially in the development of Alzheimer’s and Parkinson’s diseases. In this review, we focus on the mechanisms underlying the involvement of the P2RX7 purinoreceptor in triggering mitochondrial dysfunction during the development of neurodegenerative disorders. We also summarize various avenues by which the purine signaling pathway may trigger metabolic dysfunction contributing to neuronal death and the inflammatory activation of glial cells. Finally, we discuss the potential role of the purinergic system in the search for new therapeutic approaches to treat neurodegenerative diseases.

## Visual Abstract

**Figure:**
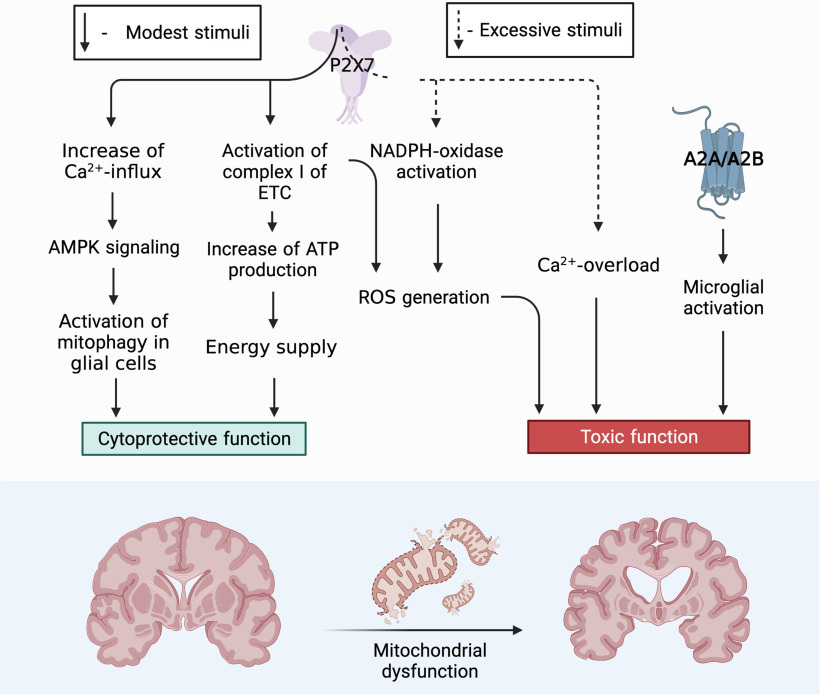

## Significance Statement

We wanted to understand the role of purinergic signaling system in the development of mitochondrial dysfunction in neurodegenerative diseases. This is important because the development of neurodegenerative diseases is closely connected with the mitochondrial dysfunction of nervous cells and purinoreceptors can by triggers in the neuroinflammations. Our results provide a guide on what the potential role of purinergic signaling in the development of neuroinflammation in Alzheimer’s and Parkinson’s diseases is. We found the dual-functional role of purinoreceptor (P2X7), which is involved in both cell proliferation and apoptotic cell death. Investigation of these mechanisms allows researchers to focus their attention on the search for pharmacological targets.

## Introduction

Although there is a number of known causative factors, mitochondrial dysfunction serves as a principle driver of neurodegenerative disorders (Mattson et al., 2008; Negroni et al., 2015; Pickrell and Youle, 2015; Abramov et al., 2017; Sukhorukov et al., 2020.; Kaur et al., 2021; Onyango et al., 2021). Mitochondria provide the cell’s energy sources and also participate in the control of secondary messenger levels such as Ca^2+^ ions and reactive oxygen species (ROS; Roger et al., 2017; Giorgi et al., 2018). Consequently, mitochondrial dysfunction facilitates a decrease in ATP production, Ca^2+^ dyshomeostasis, and ROS generation. It is known that mitochondria are the primary source of ROS and generate superoxide at the initial site in the respiratory chain under conditions of ischemia and hypoxia. ROS attack macromolecules within the neuronal plasma membrane which leads to their oxidative modification and destruction (Subramaniam and Chesselet, 2013). In the earlier stage of Alzheimer’s disease, alterations of mitochondrial dynamics and mitophagy occur, but the principle mechanisms underlying these changes have yet to be studied in detail (Tapias, 2019).

The metabolic regulation system mediated by purinergic signaling plays a separate role in the neuronal response to oxidative stress. A large amount of experimental data on the modulating effect of ROS on purinergic signaling has been accumulated (Fujita et al., 2009; Espada et al., 2010; Cieślak and Wojtczak, 2018; Maiolino et al., 2019). In particular, a modulating effect of ROS on P2XR purinergic receptor channels has been demonstrated (Mason et al., 2004), which indicates the participation of ROS not only acts as agents triggering oxidative stress but also as neurotransmitters. P2RX7 is unique among purinergic receptors since it is involved in mechanisms of neuroinflammation and is responsible for ATP-dependent cell lysis (Falzone et al., 2018).

This review aims to summarize our current understanding of the role of the P2RX7 receptor and purinergic signaling in the development of mitochondrial dysfunction in neurodegenerative diseases. An understanding of these mechanisms will make it possible to develop and supplement new and existing therapeutic approaches in the treatment of neurodegenerative disorders. Indeed, the potential of purinergic receptor mechanisms as therapeutic targets for the treatment of neurologic and degenerative diseases has already been demonstrated in several studies (Burnstock, 2006; Engel et al., 2016; Woods et al., 2016).

## The Concept of Purinergic Signaling and Mitochondrial Physiology

ATP release is regulated by the mechanism of autocrine feedback under normal physiological conditions (Corriden and Insel, 2010; Burnstock, 2015). Extracellular ATP (eATP), associated with purine and pyrimidine nucleotides, perform their functions by transmitting signals through P2 receptors. These receptors are widely represented on various cells of the body (Atkinson et al., 2002).

In the physiology of purine signaling, the following key elements are prominent: ATP release from cells in response to the activation of the cell surface receptor, autocrine activation of P2 receptors, ATP hydrolysis and the formation of adenosine by ectonucleotidases (Bagatini et al., 2018), activation of P1 receptors, and the removal and conversion of adenosine (Junger, 2011).

### Families of purinergic receptors and the biology of the P2X7 receptor

A role for the ATP molecule acting as a powerful signal activated at the cell surface via families of purinergic receptors has been previously identified (Campwala and Fountain, 2013). Based on pharmacology and function, purinergic receptors were classified into P1 and P2 families. Adenosine is an essential ligand for the P1 family, and ATP, ADP, and other ligands are for the P2 family.

**Figure 1.**
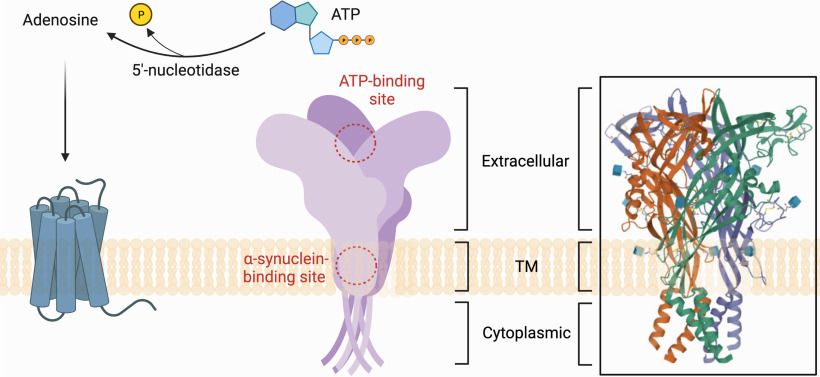
Schematic models of P2X7 receptor. Extracellular ATP is a P2RX7 receptor agonist, as well as a substrate for 5′-ectonucleotidase, which hydrolyzes ATP to adenosine and temporarily generates ADP, which is a P2Y receptor agonist. Adenosine, the product of hydrolysis of adenine nucleotides, activates adenosine receptors or P1 receptors. P2X receptors are ionotropic, and their activation opens the cation channel, which leads to cell hyperpolarization because of the outflow of K^+^ and the influx of extracellular Na^+^ and Ca^2+^. *Figure Contributions*: Alexsandra S. Zelentsova prepared the structure of P2X7 receptors and several sites for ligands. Plamena R. Angelova developed the ATP-binding site and activation of P2X7 receptor by ATP molecule.

P1 receptors include four subtypes: A1, A2A, A2B, and A3 (North and Jarvis, 2013; von Kügelgen and Hoffmann, 2016). P2 receptors are divided into two subtypes: ionotropic (P2X) and metabotropic (P2Y). Seven subtypes of ionotropic P2X receptors and eight subtypes of metabotropic P2Y receptors are known (White and Burnstock, 2006). The structural features of purinergic receptors, the architecture of the subunits of homomeric and heteromeric channels, and signal transduction mechanisms are presented in several reviews (Piirainen et al., 2011; Compan, 2012; Hattori and Gouaux, 2012; Baroja-Mazo et al., 2013; North, 2016; Kroemer and Verkhratsky, 2021; Sarti et al., 2021). The structural and schematic models of P2X7 receptor, the most studied member of P2X receptors family, are presented in Figure 1.

Purinergic P2X receptors are activated by extracellular ATP (Burnstock and Williams, 2000). All members of this family share a common structure, including the transmembrane domain, a large extracellular loop, and intracellular N and C termini (North, 2002). The sequences from the N terminus to the second transmembrane domain are relatively identical. The C terminus is specific for individual P2X receptors and includes consensus binding motifs for protein kinases and other regions that may be involved in intracellular signaling. The P2RX7 is unique in the P2RX family. First, it is activated by a relatively unusually high concentration of eATP (1 mm below the physiological concentration). Second, it allows the penetration of large molecules up to 900 Da, and additionally, it is responsible for eATP-dependent cell lysis, a response that depends on the presence of the C-terminal sequences (Falzone et al., 2018). P2RX7 is associated with lipid rafts, which depend on palmitoylation of Cys residues located in the C-terminal region (Feng et al., 2006). The binding of ATP or its agonists to P2X7 causes a rapid uptake of Ca^2+^ and exposure of phosphatidylserine (PtdSer) to the cell followed by the release of microvesicles, pore formation, and pyroptosis (Savio et al., 2018). P2RX7 mRNA is expressed by almost all cells. P2RX7 protein can be expressed but persists in the cytoplasm in an inactivated form (Benzaquen et al., 2019). The P2RX7 gene is located on chromosome *12q24* and consists of 13 exons. P2RX7-A mRNA is formed by constitutive splicing, but 12 additional transcripts can be generated by alternative splicing (Cheewatrakoolpong et al., 2005).

### The purinergic metabolome in mitochondria

Mitochondria are dynamically linked and can exchange material with one another and with other cellular organelles such as lysosomes and endoplasmic reticulum (ER; Marchi et al., 2014; Soto-Heredero et al., 2017). Mitochondrial fusion is occurred through GTF hydrolysis and is coordinated by the mitofusins MFN1 and MFN2, and the optic atrophy 1 (OPA1) protein located on the outer mitochondrial membrane (OMM; Wai and Langer, 2016). MFN2 is also present on the ER and controls binding of the mitochondria to ER (de Brito and Scorrano, 2008; Naon et al., 2016; Basso et al., 2018). OPA1 is anchored to the inner mitochondrial membrane (IMM) and is responsible for mitochondrial fusion (Delettre et al., 2000). The mitochondrial-specific lipid, cardiolipin, is attached to OPA1 and plays a critical role in inner mitochondrial membrane fusion (Ban et al., 2017; Tilokani et al., 2018). In the regulation of mitochondrial dynamics, proteolytic processing plays a key role, as reviewed by Dietz et al. (2019). Deficiency or loss of fused proteins leads to fragmentation of mitochondria (Ichishita et al., 2008; Kanazawa et al., 2008).

In mitochondrial physiology, an important role is attributed to the purinergic metabolome, which includes the linkage of purines with purinergic receptors and nucleotidases, which is regulated by purinosomes (An et al., 2008). Purinosomes are mesoscale assemblies formed to protect unstable intermediate products and increase metabolic flux during *de novo* synthesis of purines (Zhao et al., 2013). These structures are dynamic and reversible in response to the depletion of purines (Zhao et al., 2015). Their formation is cell cycle dependent, and is regulated by G-coupled-proteins agonists (GPCR) and casein kinase 2 (Chan et al., 2015). An increase in the overall number of cells containing purinosomes, positively correlates with the degree of purine deficiency in Lesch-Nyhan disease (R. Fu et al., 2015).

Purinosomes are colocalized with mitochondria, supplying the immediate demand for purines via microtubule-driven interactions (Savio et al., 2021). This spatial interaction depends on regular mitochondrial activity and is linked with the mTOR signaling pathway (French et al., 2016; Pedleyet al., 2018). A variety of kinase cascades are involved in purinosome regulation, including the PI3/AKT cascade in the mTOR signal pathway (French et al., 2013).

Mitochondrial physiology is closely associated with intracellular communication and development of different neurodegenerative diseases. It has been shown that whole mitochondrion transfer between cells confers neuroprotective activity (Hayakawa et al., 2016). Mitochondrial stress has also been demonstrated to stimulate the release of molecules such as damage-associated molecular pattern (DAMP) proteins, which generate strong proinflammatory activity (Sliter et al., 2018). PINK and PARKIN are implicated in general control over the quality of mitochondria (Y. Yang et al., 2006), and both have been observed to be mutated in familial cases of PD (Trinh and Farrer, 2013). The mitochondrion is a target for PINK1, but it breaks down very quickly in the cytosol through proteolytic pathways by cleavage of the N terminus (Kondapalli et al., 2012; Lazarou et al., 2012; Yamano and Youle, 2013). PARKIN ubiquitinates some proteins on the surface of mitochondria that are then recognized as autophagy adaptor proteins and delivered to the autophagosome (Chen and Dorn, 2013; Sarraf et al., 2013).

The functional activity of the mitochondrial network depends on protein folding. Incorrectly folded proteins in the mitochondrial matrix induce mitophagy (Jin and Youle, 2013). Nevertheless, mitochondria can form mitovesicles that protect them from mitophagy by removing misfolded proteins. Sugiura et al. (2014) described a model of mitovesicle formation using electron microscopy. Phosphorylated ubiquitin on the mitochondrial membrane can act as a signaling mechanism for common cellular responses to mitochondrial stress. Soubannier et al. (2012) described the formation of mitovesicles enriched with the oxidized protein under conditions of mitochondrial stress, while the nature of the protein load depended on the type of induced stress.

Studies on the proteome of mitovesicles have shown that it is enriched with components of catabolic pathways, electron transport chain proteins, subunits of the pyruvate dehydrogenase complex and Krebs cycle proteins, catabolism of ketone bodies, β-oxidation of fatty acids, and metabolism of neurotransmitters. Concurrently, mitovesicles lack cytosolic mitochondrial peptides, iron-sulfur clusters, proteins of ubiquinone biosynthesis, mitoribosome components, mitochondrial tRNA (mtRNA), enzymes of metabolism and replication of mtDNA, and enzymes of transcription and translation (D’Acunzo et al., 2021). Moreover, recent experimental data revealed different mitovesicles profile derivates within neurons and astrocytes (Fecher et al., 2019). Specifically, sideroflexin-5 (Sfxn5) and monoamine oxidase type B (MAO-B) are particular to astrocytes, while the homolog of the NipSnap1 protein, the OCIA domain-containing protein 2 (OCIAD2), mitochondrial calcium uniporter (MCU), and monoamine oxidase type A (MAO-A) are predominant in neurons (D’Acunzo et al., 2021).

### Mitophagy, mitochondrial homeostasis, and neurodegeneration diseases

Mitophagy plays an important physiological role in maintaining mitochondrial homeostasis (Palikaras et al., 2018), regulates such aspects of mitochondrial physiology as dynamics, biogenesis, transport, and recruitment of autophagosomes to eliminate defective mitochondria (Harper et al., 2018). Several molecular pathways of mitophagy regulated through ubiquitin dependent or independent signaling cascades are described in modern works (Khaminets et al., 2016). Ubiquitin-dependent mitophagy is regulated by PINK1 kinase according to modern experimental data (Pickles et al., 2018). At physiological conditions, PINK1 is transported to the inner mitochondrial membrane (IMM) of healthy mitochondria and is cleaved by presenilino-associated rhomboid protein (PARL) to a 52-kDa fragment that is released into the cytosol. The PINK1 fragment is then rapidly ubiquitinated and cleaved by the ubiquitin-proteasome system (UPS). However, various stimuli that cause mitochondrial damage lead to the accumulation of 63-kDa PINK1 on the outer mitochondrial membrane (OMM). PINK1 is then activated by autophosphorylation during mitophagy via PARKIN anchored to OMM and facilitating its E3 ligase activity (Palikaras et al., 2018; Ryan et al., 2021). Then, PARKIN and ubiquitin are phosphorylated, resulting in the assembly of ubiquitin-linked chains K6-, K11-, K48-, and K63- on the OMM (Heo et al., 2015). Parkin builds ubiquitin chains on damaged mitochondria for lysosomal degradation. Mitophagy mediated by the PINK1/Parkin pathway plays an important role in the mitochondrial quality control system and in the elimination of misfolded and unwanted proteins deposited in mitochondria (Cen et al., 2021).

Other ubiquitin E3 ligases such as Gp78 (M. Fu et al., 2013), SMURF1 (Orvedahl et al., 2011), SIAH1 (Szargel et al., 2016), MUL1 (Lokireddy et al., 2012), and ARIH1 (Villa et al., 2017) are also involved in the regulation of mitophagy. After localization on the surface of the OMM, these ligases generate ubiquitin chains, triggering the recursion of autophagy adapters such as optinervin (OPTN), nuclear dot protein 52 (NDP52), and p62 (Lazarou et al., 2015).

**Figure 2.**
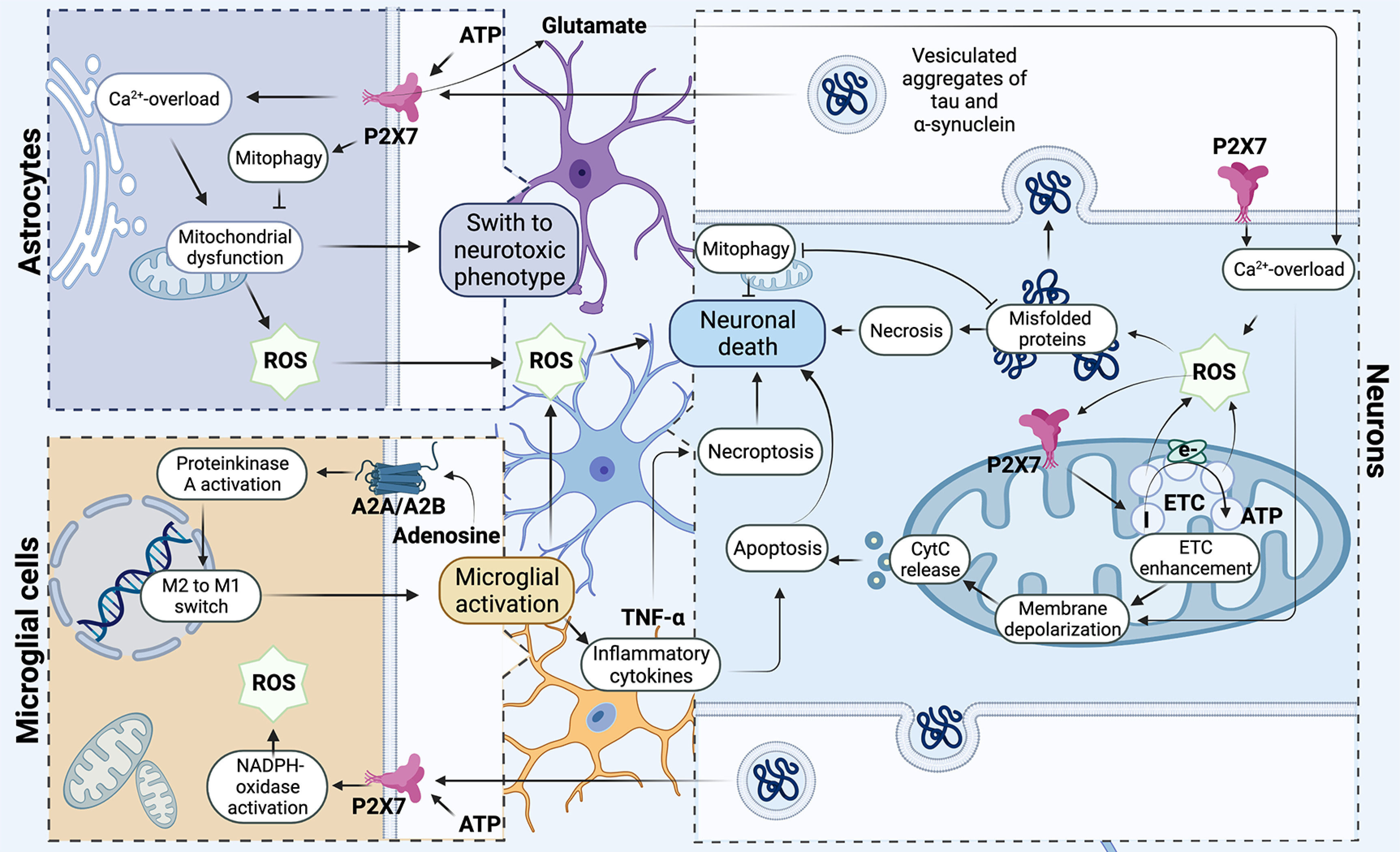
Participation of the P2X7 receptor in the development of mitochondrial degeneration in neurons. The P2X7 purinergic receptor is involved in modulating the redox potential, triggering the production of hydrogen peroxide through the mechanism of Ca^2+^ release from intracellular stores from the primary microglia. At the same time, in neurons, mitochondria are the primary source of ROS and generate superoxide at the initial site in the respiratory chain under conditions of ischemia and hypoxia. ROS initiates increase of P2X7 expression on the mitochondrial membrane and activity of Complex I, hence increasing mitochondrial polarization, which leads to an elevation of a cascade of unfavorable processes: depolarization of the mitochondrial membrane, calcium overload, and release of cytochrome *c*, which ultimately leads to cell death by apoptosis way. Under conditions of excessive accumulation of ROS, the process of incorrect aggregation of α-synuclein and tau protein triggered. Oligomeric proteins are involved in coordinating neuroinflammation via the direct interaction of extracellular oligomers with the P2X7 receptor of microglia, which triggers NADPH oxidase activation. Oligomeric proteins trigger the development of mitochondrial dysfunction, localizing in mitochondria and reducing their oxygen consumption and significantly increasing extracellular production of hydrogen peroxide by astrocytes. Purinergic receptors play a key role in the process of neuroinflammation and can trigger activation of microglia. In activated microglial cells, P2X7 is directly involved in triggering the production of TNFα cytokine that can be involved in the process of neuronal death both through apoptosis and through necroptosis; in addition, this receptor stimulates ROS production and triggers mitochondrial dysfunction. Adenosine receptors are involved in the regulation of microglial polarization. Activation of microglia closely connected with purine receptors P1 associated with G-proteins. Activation of adenosine receptor (AR) regulates the activity of protein kinase A (PKA) and participates in the switching of phenotype M1/M2 in the microglial cells. *Figure Contributions*: Alexsandra S. Zelentsova prepared the mechanism developing of neuronal death. Alexei V. Deykin developed the involvement of oligomeric proteins in the organization of neuroinflammation. Vladislav O. Soldatov performed microglial activation. Anastasia A. Ulezko developed forming ROS by mitochondrion. Alina Y. Borisova developed the pathogenetic mechanisms between microglia, astrocytes, and neuron. Veronika S. Belyaeva performed the formation of unregular folding oligomeric proteins. Marina Y. Skorkina performed the mechanisms of organization of neuroinflammation. Plamena R. Angelova developed the activation of the microglial neuron and the role of P2X7 receptor in neuroinflammation and death of neuron ny necroptosis and apoptosis.

Impaired mitophagy mechanisms are closely associated with the development of neurodegenerative diseases including Alzheimer’s disease and Parkinson’s disease (PD; Park et al., 2021). The development of PD is associated with the occurrence of mutations in the PINK1-PARKIN pathway, which inhibits mitophagy (Youle and Narendra, 2011). PARKIN mutations associated with PD prevent the recruitment of PARKIN to mitochondria and the accumulation of damaged mitochondria (Fig. 2). This increases ROS production thereby contributing to the development of PD pathologies (Pickrell and Youle, 2015).

Moreover, α-synuclein disrupts mitophagy through different signaling pathways. In neurons of PD patients, α-synuclein interacts with Miro through its N terminus and increases the level of Miro protein on OMM, which leads to delayed mitophagy. These data indicate that Miro is a target of α-Syn-associated mitochondrial damage (Liu et al., 2019). In addition, overexpression of the A53T α-synuclein mutant leads to p38 MAPK activation and induces PARKIN phosphorylation at Serine131, disrupting PARKIN function and mitophagy (Hirota et al., 2015). In a mouse model with A53T α-synuclein overexpression, mitophagy and neuronal death have been shown to increase on accumulation of α-synuclein in mitochondria (Lonskaya et al., 2013; Rojas-Charr et al., 2014).

Apparently, one of the crucial mechanisms of α-synuclein-induced mitochondrial dysfunction and increased free radical production is activation of the purinergic receptor P2X7. Obviously, α-synuclein is able to directly bind to the transmemrane domain of P2X7 (Fig. 1). The stimulatory effect of α-synuclein on P2X7 and a significant mobilization of Ca^2+^ were proven on the SHSY5Y neuronal cell model (Wilkaniec et al., 2017).

Direct involvement of the P2X7 purinergic receptor in increased oxidative stress through stimulation of this receptor by extracellular α-synuclein was shown on microglial cells (Jiang et al., 2015). Reduced mitochondrial respiration through mitochondrial depolarization and disruption of mitochondrial Complex I activity followed by increased free radical production are hypothesized to be key molecular events activated by α-synuclein (Devi et al., 2008; Chinta et al., 2010; Reeve et al., 2015). Extracellular α-synuclein induces mitochondrial depolarization with a subsequent increase in the level of mitochondrial superoxide as well as dysregulation of mitochondrial redox homeostasis. Moreover, this effect of α-synuclein largely depends on the activation of P2X7 that induces the Panx-1 recruitment and the formation of pores permeable to large molecules up to 900 Da in size (Wilkaniec et al., 2017).

AMPK is a key P2X7R signaling modulator that triggers mitophagy and mitochondrial division in microglia. On microglial cells, the mechanism of AMPK activation through ROS and calcium signaling pathways during P2X7R stimulation, leading to mitochondrial division, induction of mitophagy, and activation of the nuclear transcription factor TFEB has been proven (Sekar et al., 2018). AMPK regulates the activity of PINK and PARKIN and thus participates in the initiation of mitophagy (Herzig and Shaw, 2018). AMPK regulates PINK and PARKIN activity and thus participates in the initiation of mitophagy (Herzig and Shaw, 2018). Notwithstanding, сalcium signaling activated by P2X7R stimulation may be the molecular mechanism for the α-synuclein-dependent decrease in AMPK activity. The negative effect of P2X7R predominantly involves lysosomal disturbances in microglial cells (Takenouchi et al., 2009; Sekar et al., 2018). However, a study by Wilkaniec et al. (2020) demonstrated inhibition of AMPK activity followed by inhibition of Ulk-1 under conditions of α-synuclein-dependent P2X7 stimulation in nerve cells. It is possible that various cell types can activate different cellular pathways on P2X7 stimulation depending on the receptor isoform (Kaczmarek-Hajek et al., 2018). Thus, mitophagy is the central mechanism in maintaining cellular homeostasis and has a neuroprotective function that protects neurons from the accumulation of defective proteins.

## Purinergic Signaling and Redox Homeostasis of the Cell

Purinergic signaling affects the activity of antioxidant enzyme systems, contributing to a change in the redox potential of cells (Piacenza et al., 2019; Coutinho-Silva and Savio, 2021). Reactive oxidants regulate physiological and pathophysiological processes by acting as signaling molecules aimed at cell division and their programmed death (Torres et al., 2013), but uncontrolled production has been shown to contribute to the development of oxidative stress (Rana, 2021).

### P2X7 receptor-like inductor of oxidative stress

The P2X7 receptor is involved in modulating the redox potential and can act as an inducer of production of hydrogen peroxide (H_2_O_2_) through the mechanism of Ca^+2^ release from intracellular depots in the primary microglia of transgenic mice with Alzheimer’s disease. These results were confirmed using P2X7 inhibitors (polyphosphonamides and oxidized ATP), which blocked the production of H_2_O_2_ and P2X7 agonists (BzATP) that stimulated ROS through the activation of NADPH oxidase (Parvathenani et al., 2003). Also, the P2X7 agonist BzATP was shown to stimulate the phosphorylation of MAPK (Pfeiffer et al., 2007). The work of Zhang and colleagues demonstrated the accumulation of intracellular ROS and the subsequent activation of the NLRP3 inflammasome in macrophages from mice of the J774.1 line when the P2X7 receptor was activated (Zhang et al., 2021).

### ROS and the sensitivity of Krebs cycle enzymes

The inactivation of enzymes of the citric acid cycle is one of the negative effects of ROS. Аconitase and α-ketoglutarate dehydrogenase complex (α-KGDHC) are particularly sensitive to oxidative stress (Sadek et al., 2002). Aconitase is most susceptible to ROS which is related to the sulfur-iron complex (Tretter and Adam-Vizi, 2000). α-KGDHC is closely associated with the inner mitochondrial membrane through Complex I of the respiratory chain, and may be a target for ROS because of the close spatial proximity to sites generating ROS (Berndt et al., 2012). Concomitantly, α-KGDHC can also generate H_2_O_2_ itself and is a source of reactive oxygen species.

A previous study also demonstrated the production of hydrogen peroxide by isolated α-KGDHC with coenzyme A (HS-CoA) and thiamine pyrophosphate in the absence of nicotinamide adenine oxidized (NAD; Tretter and Adam-Vizi, 2004). In contrast, NADH stimulated the formation of H_2_O_2_ by α-KGDHC in the absence of α-ketoglutarate or HS-CoA in the medium (Tretter and Adam-Vizi, 2004).

α-KGDHC is considered one of the key enzymes which limits the rate of the Krebs cycle; accordingly, a decrease in the activity of this enzyme complex initiates a cascade of unfavorable processes: depolarization of the mitochondrial membrane, calcium overload, and release of cytochrome *c*, which ultimately leads to cell death (Huang et al., 2003). Pathophysiological reactions are triggered within the mitochondria of neurons with reduced activity of the α-KGDHC which faces a high demand for ATP in the cell. Calcium is not sufficiently exported out of the cell into the extracellular space when the cytosolic level of ATP is low, as a result, its concentration increases, which leads to the overloading of mitochondria with calcium, which against the background of reduced activity of the α-KGDHC can initiate apoptosis. This cascade has also been described in the abundant loss of dopaminergic neurons in the substantia nigra (SN) of patients with Parkinson’s disease (Fiskum et al., 2003).

### Oxidative stress as a major factor in neurodegenerative disease

ROS-induced oxidative stress is considered by researchers as a major factor in the pathogenesis of Alzheimer’s disease (Cenini and Voos, 2019). In Alzheimer’s disease, the reduction in α-KGDH activity ranges from 25% to 75%; at the same time, there is a correlation with cognitive decline (Boveris and Navarro, 2008). In experimental animal models, as well as in patients with Alzheimer’s disease, a close relationship between oxidative stress and mitochondrial dysfunction has been observed. Transgenic mice with overexpression of human *APP* (Tg *mAPP* mice) and progressive accumulation of amyloid-β (Aβ) peptide in synaptic mitochondria exhibited the development of mitochondrial synaptic dysfunction as a result of impaired respiratory activity and mitochondrial axonal transport (Du et al., 2010). A 3xTg-AD mouse model shows that disruption of mitochondrial bioenergetics, together with increased levels of oxidative stress, represents early phenomena that occur before the observed development of Aβ plaques (Hauptmann et al., 2009; Yao et al., 2011).

## Some Aspects of Neuroinflammation

### Exosomes and neuroinflammation

In recent work, extracellular vesicles have been recognized as playing an essential role in the development of neurodegenerative diseases. Vesicles are a heterogeneous family of endocytosed microvesicles originating from cell membranes circulating in the biological fluids of the organism as a result of the separation of membrane material from any cell type in the body (Paolicelli et al., 2019). Extracellular vesicles perform a regulatory role by an endocrine-like mechanism consisting of the modulation of gene expression in cells located remotely from each other (Budnik et al., 2016; Krämer-Albers and Hill, 2016). Specifically, the role of mobile vesicles produced by microglial cells has been established in the development of cytokine-mediated inflammatory responses in different parts of the brain (Frühbeis et al., 2013). Exosomes are involved in the development of neurodegenerative pathophysiological processes in nervous tissue and transport. Such proteins include α-synuclein, τ and β-amyloid, and pathogenic prion proteins (Ngolab et al., 2017; Y Wang et al., 2017; Zheng et al., 2017).

Exosomes together with purinergic receptors play a key role in the process of neuroinflammation and can trigger apoptosis (Chen et al., 2014). Extracellular ATP is a stimulator of vesiculation in microglial cells bearing P2X7 purinergic receptors. In a mouse model with subclinical inflammation, it was shown that extracellular vesicles released by ATP-stimulated microglia cause a strong inflammatory reaction in glial cells *in vitro* and can propagate an inflammatory response among microglia (Verderio et al., 2012).

In a study by Drago et al. (2017), ATP-stimulated vesiculation of microglial cells (1 h in 1 mm ATP) was shown. Several enzymes necessary for glycolysis (glucose-6-phosphate isomerase), lactate formation (lactate dehydrogenase, malate dehydrogenase), plus an enzyme of the oxidative branch of the pentose phosphate pathway (transketolase), an enzyme of glutamine metabolism (glutamate dehydrogenase 1) and an enzyme of fatty acid synthesis (acetyl-CoA carboxylase-β) were found in the composition of ATP-stimulated exosomes. ATP-stimulated exosomes also contain proteins of the cytoskeleton and chaperones that interact with the P2X7 C terminus (Gu et al., 2009). In addition, microglial exosomes can function as independent metabolic units and have the potential to increase glucose-based energy outside of mitochondria in recipient cells (Iraci et al., 2017).

### The P2RX7 receptor of exosomes as a signal for tau protein spread

Tau protein is known to be associated with microtubules: it interacts with α and β-tubulin subunits helping microtubule assembly. Six isoforms of tau protein have been described which are formed by alternative mRNA splicing and phosphorylation of multiple sites (Kumar et al., 2015). Phosphorylation can lead to oligomerization and the resulting aggregates are implicated in cell-mediated transport which underlies the basis of disease spreading in tauopathies (Kundel et al., 2018).

In a recent study, it was shown that insoluble aggregates of tau proteins can induce ROS production by activation of NADPH oxidase in a calcium-dependent way (Esteras et al., 2021). Furthermore, the activation of NADPH oxidase in combination with membrane-active properties of tau protein aggregates causes neuronal death (Olguín-Albuerne and Morán, 2015).

The main role in the distribution of pathologic tau protein is attributed to exosomes released by microglia during the development of Alzheimer’s disease (AD) (Asai et al., 2015). Among the many constituent exosome molecules, the researchers focused on P2X7 (P2RX7) purinoreceptors which are predominantly expressed in microglia and represent an ATP-induced Na^+^/Ca^2+^ channel (Kaczmarek-Hajek et al., 2018). The P2RX7 receptor is thought to have a central role in the progression of AD disease based on the increased expression of P2RX7 in the proximity of amyloid-β (Aβ) in the amyloid plaques of AD patients and in animal models of AD (Parvathenani et al., 2003; McLarnon et al., 2006).

Treatment of the BV2 cell line with α-synuclein increases the secretion of exovesicles expressing the MHC Class II and TNF-α molecules on the surface as well as inducing neuronal apoptosis (Chang et al., 2013). In the case of AD, it has been shown that the Aβ40 and Aβ42 species involved in amyloid formation are present in exosomes (Rajendran et al., 2006). Microglial cells modulate the clearance of Aβ because of its internalization and degradation. Microvesicles from microglia in the presence of excess amyloid are neurotoxic and also have myelotoxic effects (Agosta et al., 2014). Moreover, it has been described that inflammatory microglia release miRNA-enriched microvesicles capable of regulating the level of synaptic proteins in recipient neurons leading to the loss of excitatory synapses, indicating a new mechanism by which microglial cells can mediate synaptic changes in neurodegeneration (Prada et al., 2018).

Exosomes associated with microglia seem to play a role not only in the regulation of synaptic proteins and Aβ levels but also in the spread of tau pathology. In two different mouse models of AD, tau, a neuronal protein associated with microtubules that abnormally accumulates in degenerating neurons, was selectively absorbed by microglia (Guo et al., 2021). Nevertheless, microglia also released tau protein along with exosomes which were captured by cortical neurons thereby contributing to the spread of tau pathology (Vogels et al., 2019). Exosomes can participate in tau transmission demonstrating a certain amount of tau protein is released from neurons through exosomes and partly through synaptic activity (Y. Wang et al., 2017). Depletion of microglia or of the production of exosomes led to a decrease in the spread of mutant tau (Asai et al., 2015). Recent publications have presented data indicating that the spread of tau protein occurs together with low-density lipoproteins, receptor-like proteins, or proteoglycan heparan sulfate (Puangmalai et al., 2020; Rauch et al., 2020).

Pharmacological blockade of P2RX7 by GSK1482160 at an early stage in P301S mice significantly reduced tau accumulation in the hippocampus and exosomes as well as improved hippocampal memory (Ruan et al., 2020). Moreover, the ATP-induced secretion of exosomes from mouse microglia was significantly suppressed by the P2RX7 GSK1482160 inhibitor, whereas it did not affect the secretion of tau or exosomes of primary mouse neurons or astrocytes *in vitro*. These results indicate the functional role of P2RX7 in the secretion of microglial exosomes and the progression of tau pathology (Ruan et al., 2020).

The stimulation of the P2X7R receptor has a double effect in AD: activation of a nonamyloidogenic neuroprotective pathway and/or overactivation of glial cells causing an excessive proinflammatory response (Martin and Delarasse, 2018; Martin et al., 2019b). The work of Sanz and colleagues demonstrated the release of IL-1β by Aβ plaques during the activation of P2X7R (Sanz et al., 2009). It was found that short-term stimulation of the receptor activates nonamyloidogenic proteolytic cleavage of the precursor protein of the amyloid peptide: α-secretase cuts the APP in the Aβ peptide sequence, which prevents the formation of neurotoxic Aβ peptides and produces a soluble fragment of sAPPα, which is neurotrophic and has neuroprotective effects (Delarasse et al., 2011; Darmellah et al., 2012; Ortega et al., 2013; Heneka et al., 2015; Miras-Portugal et al., 2015; Sáez-Orellana et al., 2016; Hunter et al., 2018; Illes et al., 2019; Martin et al., 2019a). Consequently, activation of P2X7R may exhibit neuroprotective action.

The effect of genetic knock-out of P2X7R in *TgAPP/PS1* mice was evaluated to study the role of P2X7R in the development of AD. Using this model, it was revealed that P2X7R deficiency reduces the pathologic effects of the amyloid protein, improves synaptic plasticity in the hippocampus, and normalizes memory. This effect is realized through the recruitment of immune cells in the CNS (Martin et al., 2017; Martin et al., 2019b). The absence of P2X7R reduces the set of cytotoxic CD8 T-lymphocytes which correlates with a decrease in the level of CCL3. The expression of CCL3 and its CCR5 receptor is elevated in the brains of patients with AD and AD mouse models. In these mice, overexpression of CCR5 leads to cognitive impairment. Conversely, inhibition in the hippocampus improves memory (Zhou et al., 2016; Martin and Delarasse, 2018).

### α-Synuclein and neuroinflammation

α-Synuclein is mainly located at presynaptic terminals in the brain and participates in vesicular transport, neurotransmitter release, and synaptic plasticity (Stefanis, 2012). Furthermore, the localization of α-synuclein in the nucleus and the membranes of the endoplasmic reticulum-associated with mitochondria has been previously described (Surguchov, 2015). α-Synuclein in monomeric form takes part in synaptic signal transduction: it binds to F0/F1-ATP synthase and increases the ATP synthesis rate in mitochondria under physiological conditions (Ludtmann et al., 2016). Tau protein joins microtubules and is involved in their stabilization and growth processes (Y. Wang and Mandelkow, 2016).

Oligomeric α-synuclein is involved in coordinating neuroinflammation via the direct interaction of extracellular oligomers with the P2X7 receptor of microglia (Hou et al., 2019), which triggers NADPH oxidase activation (Q. Wang et al., 2015). On this basis, it was shown that inhibition of NADPH oxidase by apocynin prevents learning and memory disorders in a model of Parkinson’s disease (PD) in mice (Hou et al., 2019).

Oligomeric α-synuclein binds to F0/F1-ATP synthase causing oxidation of its β-subunit and also lipid peroxidation of the mitochondrial membrane, which in combination with calcium overload, leads to the formation and opening of additional mitochondrial permeability channels and, as a consequence, cell death (Ludtmann et al., 2018). α-Synuclein fibrils also cause neurotoxicity and cell death through activation of nitric oxide synthase (NOS), resulting in DNA damage and activation of polymerase-1 (PARP-1). PARP-1 accelerates α-synuclein fibrillation (Kam et al., 2018). According to the experimental data presented in several works, oligomeric α-synuclein is involved in the initiation of apoptosis (Angelova et al., 2015), necrosis (Reeve et al., 2015), and ferroptosis (Angelova et al., 2020).

Experimental molecular docking data confirm the interaction between α-synuclein and the BH3 domain of BAX, and it has been suggested, that the α-synuclein-BAX complex migrates into mitochondria and initiates apoptotic cell death (Dewson and Kluck, 2009; Chakrabarti et al., 2020).

## Purinergic Signaling Pathway and Mitochondrial Disruption

Mitochondrial dysfunction of neurons, astrocytes and microglia is a key factor in the development of neurodegenerative diseases. One of the central signaling mechanisms for the development of mitochondrial dysfunction in neurons and neuroglia is the mobilization of intracellular calcium which can be triggered by the P2X7 purine receptor, as well as through adenosine receptors (ARs). The P2X7 receptor is predominantly localized on the plasma membrane of astrocytes and microglia (Tsuda, 2017). In neurons, its localization on the membrane has not been proven; however, there is evidence that neuronal P2X7R exists as false immunologic signals, but also as potentially operational subunits that regulate neuronal function (Anderson and Nedergaard, 2006; Sperlágh and Illés, 2006). Data have been presented that functional P2X7R is also expressed in immortalized dopaminergic neurons (SN4741 cells) obtained from the substantia nigra of transgenic mouse embryos and performs a function in neuronal differentiation (Jun et al., 2007). In several studies, P2X7R intracellular localization was indicated on the nuclear membrane (Atkinson et al., 2002), mitochondria (Sarti et al., 2021), and intracellular phagosome (Kuehnel et al., 2009). P2X7Rs are thought to induce apoptosis in glial cells through caspase activation and necrosis (Zhao et al., 2018). P2X7Rs control microglial activation and proliferation, potentially leading to a destructive cycle of neuroinflammation and neurodegeneration (Monif et al., 2010).

The P2X7 purinergic receptor is involved in modulating the redox potential, triggering the production of hydrogen peroxide through the mechanism of Ca^2+^ release from intracellular stores (Piacenza et al., 2019; Coutinho-Silva and Savio, 2021). Excessive accumulation of ROS against the background of dysregulation of the antioxidant system leads to damage to enzymes of the Krebs cycle (aconitase, α-CTGD), oxidation of proteins, DNA, and lipids, thereby disrupting mitochondrial energy which ultimately acts as a pathogenetic mechanism in the development of neurodegenerative diseases (see Fig. 2) and predetermines the aging of the brain and its performance (Rana, 2021).

Mitochondrial dysfunction of astrocytic and microglial cells is associated with their activation. Disruption of calcium homeostasis, overproduction of ROS, and triggering of astrocyte death cascades have all been described in activated astrocytes (Gollihue and Norris, 2020). Oligomeric α-synuclein acts as a factor in the activation of astrocytes and triggers the development of mitochondrial dysfunction, localizing in mitochondria and reducing their oxygen consumption (Braidy et al., 2013). It was found that the accumulation of α-synuclein aggregates in the trans-region of the Golgi complex also disrupts mitochondrial dynamics and leads to fragmentation of mitochondria (Verkhratsky et al., 2016). Fibrillar α-synuclein induces mitochondrial dysfunction of astrocytes and significantly increases the extracellular production of hydrogen peroxide by cells (Chavarría et al., 2018). Moreover, astrocytes can be activated by fragments released from microglia, which are viewed as neurotoxic signaling mediators that inhibit neuronal mitochondrial respiration, significantly increasing neuronal death (Joshi et al., 2019). The process of astrocytic mitophagy is important for the survival of neurons, and astrocytes are directly involved in the spheroid-mediated transmytophagy of dopaminergic neurons. It has been experimentally confirmed that damaged mitochondria during degeneration of dopaminergic neurons are preserved in spheroids where the mitophagy process is initiated but not completed, and subsequently, these spheroids penetrate astrocytic cells and undergo further degradation (Morales et al., 2020, 2021). Consequently, neuron-astrocyte transmitophagy is crucial for preventing the release of mitochondria into the extracellular environment during neuronal death. In addition, astroglia activated by microglia have been found in the brains of patients with different neurodegenerative diseases (Hickman et al., 2018), including PD (Banati et al., 1998), AD (Maat-Schieman et al., 1994), and Huntington’s disease (Tai et al., 2007). Researchers point to the existence of two phenotypes of microglial cells: proinflammatory M1 and neuroprotective M2 types (Kwon and Koh, 2020). Microglial activation is also known to be associated with P1 purine receptors connected with G-proteins (see Fig. 2).

The impaired P2X2 receptor and purinergic signaling across different neurodegenerative diseases are summarized in Table 1.

**Table 1:** Receptors P2X2 family and neurodegenerative diseases

Receptor	Disease/organism	Brief overview	Reference
P2X7 P2X4	Parkinson’s disease Alzheimer’s disease	P2X7R expression is increased on microglia, astrocytes, and oligodendrocytes in neuroinflammatory conditions in the CNS. Activation of P2X7R by increasing the concentration of extracellular ATP released from damaged brain cells, promotes microglia activation and proliferation, and directly promotes neurodegeneration by inducing microglia-mediated neuronal death, glutamate-mediated excitotoxicity, and NLRP3 inflammatory activation, leading to initiation, maturation, and release of proinflammatory cytokines and generation of reactive oxygen species and nitrogen forms.	Territo and Zarrinmayeh (2021)
P2X7 P2X4	Parkinson’s disease Alzheimer’s disease	Activation of microglia leads to increased expression of P2X4 and P2X7 and suppression of P2Y12 receptor expression.	Di Virgilio et al. (2017)
P2X7 P2X4	Parkinson’s disease Alzheimer’s disease	An indicator of microglia activation of the proinflammatory M1 phenotype is the high expression levels of P2X4 and P2X7 receptors.	Burnstock (2006)
P2X7 A2A	Parkinson’s disease Alzheimer’s disease	Adenosine and ATP are modulators of neuroinflammatory reactions, oxidative stress, and cell death through activation of A2A and P2X7 receptors, respectively. ATP-dependent activation of P2X7Rs induces necrosis by promoting the formation and opening of nonspecific membrane pores, leading to loss of intracellular content and activation of the caspase pathway, causing apoptosis in glial cells.	Illes et al. (2019)
А2А	Huntington’s disease	The adenosinergic pathway plays a significant role in the etiology and progression of BC especially through the A2A receptor, as observed in patients and animal models.	Ollà et al. (2020)
P2X7R	Amyotrophic lateral sclerosis	Inflammation and autophagy play a crucial role in the pathogenesis of ALS, while several studies have identified a role for P2X7R in the pathogenesis.	Volonté et al. (2016)
P2Y6	Alzheimer’s disease, multiple sclerosis, Parkinson’s disease, stroke, frontotemporal dementia, and CNS tumors	Microglia expresses purinergic P2Y6 receptors. P2Y6R acts as a regulator of inflammation and phagocytosis. Stimulation of this receptor by its endogenous ligand UDP can trigger the production and release of a large number of cytokines and chemokines.	Anwar et al. (2020)
P2YX7	Parkinson’s disease Huntington’s disease	Changes in the expression and activity of purinergic receptors such as P2YX7 have been noted, suggesting a potential role for this system in the etiology and progression of the disease.	Oliveira-Giacomelli et al. (2018)
P2X7R	Alzheimer’s disease	Activation of P2X7R leads to the opening of nonselective cation channels the constant activity of which causes depolarization of mitochondria and plasma membrane, plasma membrane pore formation, plasma membrane ballooning, and production of reactive oxygen species. Similarly, P2X7R activation increases the release of TNF-α, IL-18, and IL-6 and induces apoptotic cell death, making P2X7R an attractive therapeutic target to reduce inflammation and ATP-induced apoptosis via P2X7R antagonism.	Woods et al. (2016)
P2X4R	Parkinson’s disease	Current thinking suggests that activation of microglia and the subsequent release of inflammatory factors, including interleukin-6 (IL-6), are involved in the pathogenesis of PD. The P2X4 receptor (P2X4R) is a member of the P2X superfamily of ATP-activated ion channels. Following chronic constrictor injury (CCI), repeated administration of a P2X4R antagonist reduces IL-6 levels, implying that P2X4R can modulate neuroglial activation and IL-6 release. It has been suggested that P2X4R may play an important role in the pathogenesis of PD by damaging dopamine neurons through some specific mechanisms.	Jurga et al. (2017)
P2Y6	Parkinson’s disease	UDP binding by P2Y6R leads to the activation of various biochemical pathways depending on the disease context and pathologic environment. P2Y6R normally stimulates phagocytosis. P2Y6R can participate in LPS-induced neuroinflammation. Blocking P2Y6R is seen as a therapeutic target for the treatment of PD and AD patients by inhibiting microglia-induced neuroinflammation.	X. Yang et al. (2017)

Extracellular ATP is cleaved by ectonucleotidases with the formation of AMP which under the influence of the substrate ecto-5′-nucleotidase (CD73) decomposes to extracellular adenosine. Adenosine can be released from cells into the extracellular environment and act on P1 receptors located in neurons and is considered like a neuromodulator. So far, four subtypes of P1 families have been found: A1, A2A, A2B, and A3. Subtypes A1 and A3 bind to G_i_-coupled GPCRs thereby inactivating adenylate cyclase and decreasing intracellular cAMP levels. A2A and A2B bind to G_s_-coupled GPCRs, activating adenylate cyclase and increasing intracellular cAMP levels (Yakovlev et al., 2022). Therefore, activation of the adenosine receptor (AR) regulates the activity of protein kinase A (PKA). In addition, protein kinase C (PKC) can be activated through the mobilization of intracellular calcium-mediated by the A2B receptor (Alnouri et al., 2015). Mitochondrial homeostasis in neurons is a key factor in preventing neurodegeneration (Li et al., 2020). Despite adenosine receptors being activated in response to extracellular stimuli, they are also found in mitochondria (Valenzuela et al., 2016; Melser et al., 2017), where they are involved in controlling oxidative stress and mitochondrial performance. Adenosine receptors are involved in the regulation of microglial polarization. A2AR activation leads to increased release of nitric oxide by activated microglia. This study demonstrated that the effect was dependent on the presence of astroglia although both A2AR expression and NO synthase II immunoreactivity were observed in microglia only (Saura et al., 2005).

An important aspect to consider in neurodegenerative disease development is that vesicular transport is used as a means of intercellular communication. Researchers have assigned exosomes, together with receptors of the purinergic signaling system, a key role in the formation of neuroinflammation and the initiation of apoptosis. Extracellular ATP stimulates the vesiculation of microglial cells carrying on their surface P2X7 receptors of the purinergic signaling system (Verderio et al., 2012); these experimental studies have thus provided evidence of the P2RX7 receptor in the progression of AD disease.

P2X7 is directly involved in the regulation of various cell death pathways, such as apoptosis, pyroptosis, necrosis, and autophagy, by the fact that this receptor can regulate cell death via caspase-8 and caspase-9, stimulating AFC production, mitochondrial dysfunction (see Fig. 2), cytochrome *c* release, and caspase-3/7 activation (Bidula et al., 2019). The involvement of the P2X7 receptor in triggering necroptosis is mediated by its long-term stimulation on glial cells and their release of TNFα (Salas et al., 2013).

Necroptosis, as a type of programmed cell death of necrosis independent on caspases, is activated in response to activation of death receptors and depends on activation of receptor-interacting kinase 3 (RIPK3; activated by RIPK1) and pseudokinase, which acts as the main effector of necroptosis (MLKL). MLKL oligomerizes under the influence of RIPK3 and moves to the lipid rafts of the plasma membrane with pore formation, causing influx of Na^+^ ions and cell death (Su, 2015; Grootjans et al., 2017; Liu, 2019). It has been proven that RIP3 leads to a switch in cellular metabolism which is accompanied by an increase in the production of mitochondrial ROS and ultimately of cell death. Necroptosis is characterized by rapid membrane degradation accompanied by the release of intracellular contents, that is, damage-associated molecular patterns (DAMPs) such as highly mobile group 1 protein (HMGB1), heat shock proteins, DNA, and RNA, which activate the pattern recognitions receptors (PRRs) to further stimulate the inflammatory response (Negroni, 2015).

Various triggers, including TNF, Fas, TNF-associated apoptosis-inducing ligand (TRAIL), interferon (IFN), lipopolysaccharide, DNA damage, viral infection, and cancer cell suppressant drugs, are believed to trigger necroptosis in cells (Holler et al., 2000; Jouan-Lanhouet et al., 2012; Grootjans et al., 2017; Xu et al., 2021). Fas, TNF-induced, and TRAIL-induced necroptosis requires the activity of RIPK1 kinase, which has several domains and can activate various cell death pathways (Cai et al., 2014; H Wang et al., 2014; Hribljan et al., 2019). Inhibitor of necroptosis is caspase-8. Necroptosis represents, a tightly regulated process, the malfunction in the regulation of which is associated with the development of human diseases, which are accompanied by cell loss and inflammatory reactions (Chen et al., 2019).

Thus, necroptosis is involved in the development of neurodegenerative diseases such as AD, ALS, PD. Increased levels of markers of necroptosis, including RIPK1, MLKL, necrosome complex, and MLKL oligomer, in these diseases have been noted (Zheng-gang and Jiao, 2020). Because of these findings, the possibility of inhibiting necroptosis by interfering with and reducing the activity of necrosome components is being considered as new therapeutic targets for treatment (Caccamo et al., 2017).

## Purinergic Receptors as Therapeutic Targets for the Treatment of Alzheimer’s and Parkinson’s Diseases

Purinergic signaling pathways are now considered as therapeutic targets in the treatment of various pathologies. In the difficult treatment of neurodegenerative diseases, successes can be realized, provided that new therapeutic targets are identified.

### P2X7 receptors as therapeutic targets

The P2X7 receptor is promising as a therapeutic target in the development of Parkinson’s disease. A study by Oliveira-Giacomelli et al. (2019) found that ATP and its metabolites were extensively released in an animal model of Parkinson’s disease induced by 6-hydroxydopamine (6-OHDA). Previous work has examined the effects of pharmacological antagonists on the P2X7 receptors in preventing as well as altering hemi-Parkinson’s behavior and dopaminergic deficiency. The P2X7 receptor antagonist brilliant blue G (BBG) at a dose of 75 mg/kg restored the dopaminergic nigrostriatal pathway in rats (Carmo et al., 2014; Y. Wang et al., 2017; Oliveira-Giacomelli et al., 2019). Therapeutic strategies in the treatment of Alzheimer’s disease are also aimed at suppressing neuroinflammation, organized with the participation of microglial cells.

Microglial cells and astrocytes overexpress the P2X7R receptor which has been shown to be responsible for NLRP3 (cryopyrin, part of the NOD-like receptor family) inflammasome activation and promotes the release of the proinflammatory cytokine interleukin-1β (IL-1β). IL-1β was in turn found in the brains of AD patients and in a mouse model with developing amyloid lesions (TgAPP/PS13 transgenic mice; Marcoli et al., 2008; Bhaskar et al., 2010; Cervetto et al., 2013; Ficker et al., 2014; Di Virgilio et al., 2017; Martin et al., 2017; Martin and Delarasse, 2018; Martin et al., 2019a).

At present, the idea of developing a drug that can act on the NMDA and P2X7 receptors simultaneously would present a useful and promising treatment strategy for Alzheimer’s disease. Both receptors exhibit neuroprotective and cytotoxic effects that make them ideal candidates not only for stopping the progression of the disease but also for alleviating symptoms. Hence, the most appropriate antagonist would be a compound that exhibits inhibitory activity in the low micromolar range, thus preserving the physiological function of the receptors while inhibiting them when overactivated. Karoutzou and colleagues used amino adamantyl carbohydrazide as a putative dual inhibitor. These investigators also synthesized, characterized, and evaluated several new adamantyl carbohydrazides as potential dual NMDAR and P2X7R antagonists. Three derivatives showed low micromolar activity as P2X7R antagonists, and only one compound, designated “9g,” showed some activity as an NMDA receptor antagonist. New compound 9g for dual P2X7 and NMDA receptors with low inhibitory potenciec has been designed (Karoutzou et al., 2018).

### The P2Y6 receptor

PD induced by 1-methyl-4-phenylpyridine (MPP) is known to increase UDP/P2Y6R levels on SH-SY5Y-derived neuronal cells. Pharmacological inhibition of P2Y6R or knock-down of P2Y6R using small interfering RNA (siRNA) prevented MPP-induced increases in levels of ROS, superoxide-anions, inducible nitric oxide synthase (iNOS), malondialdehyde (MDA), and superoxide dismutase 1 (SOD1). Apyrase and MRS2578 have also been investigated as potential pharmacological inhibitors (Qian et al., 2018).

MRS2578 being a selective P2Y6 receptor antagonist prevents the death of dopaminergic neurons in SH-SY5Y-derived neurons both *in vitro* and *in vivo* in rat substantia nigra. In addition, authors found that both treatments were accompanied by a decrease in microglial activation in the substantia nigra. Thus, antagonism of P2Y6 receptors has clear neuroprotective effects (Oliveira-Giacomelli et al., 2019).

### A2A receptors

There is a wide range of pharmacotherapeutic agents available for the treatment of Parkinson’s disease. These include dopamine precursors with peripheral dopa decarboxylase inhibitors, dopamine agonists, cholinolytics, monoamine oxidase B (MAO-B) inhibitors, and catechol-o-methyltransferase (COMT) inhibitors. Nevertheless, the gold standard of treatment is still levodopa/carbidopa combination therapy, because levodopa has been seen to provide lasting improvement in mobility and carbidopa helps maintain appropriate levels of levodopa in the substantia nigra (SN; Hayes, 2019; Armstrong and Okun, 2020; Sahoo et al., 2020). However, the therapeutic effects begin to wane and are gradually outweighed by side effects such as on-off fluctuations with or without dyskinesia, clinically characterized by the absence and recurrence of motor symptoms with continued use of this combination (Armstrong and Okun, 2020; Sahoo et al., 2020). In addition to existing therapies, researchers have suggested the inclusion of drugs aimed at inhibiting A2A purinergic receptors, which inhibit the amplification of signaling through A2A receptors and reduce signaling through D2 dopaminergic receptors (Sahoo et al., 2020; Singh et al., 2021). These changes explain the positive effect of A2A receptor antagonists on enhancing motor function without progression of levodopa-induced dyskinesias (Fuxe et al., 2015; Navarro et al., 2016). Accordingly, it is logical to use selective A2A receptor antagonists such as istradefylline as additional agents to alleviate these.

Istradefylline (KW-6002) is a xanthine-based compound with increased selectivity for A2A receptors against A1 receptors (Poewe et al., 2012), and in a rat model of lipopolysaccharide (LPS)-induced neuroinflammation, it showed an anti-inflammatory effect of this compound (Gołembiowska et al., 2013). In addition to istradefylline, several powerful and highly selective inhibitors of adenosine A2A receptors have been developed (Shook and Jackson, 2011). The drugs istradefylline, preladenate, and tozadenate have been tested in phase 2b and phase 3 clinical trials and were potent and highly selective A2A receptor antagonists with good CNS penetration (Brooks et al., 2010; Pawsey et al., 2013; Pinna, 2014; LeWitt et al., 2020). Preladenate, however, did not make it to the end of phase 3 clinical trials in the treatment of PD because the compound did not outperform placebo in reducing the “off” state, and tozadenate was excluded from the phase 3 clinical trial in the  year 2018 because of serious side effects, hematologic toxicity such as agranulocytosis (Hauser et al., 2014, 2015; Pinna, 2014; Pinna et al., 2018; LeWitt et al., 2020).

A2A receptor antagonists can also directly or indirectly affect microglia and inflammatory processes. Gyoneva et al. (2014) processed brain slices of mice injected with 1-methyl-4-phenyl-1,2,3,6-tetrahydropyridine (MPTP) and concluded that preladenate adequately promotes microglial responses to tissue damage as well as enhancing the therapeutic effect of low dose l-DOPA (Gyoneva et al., 2014).

Information on preclinical and clinical trials of substances showing antagonism to purine receptors is presented in Table 2.

**Table 2:** Purine receptor antagonists in Parkinson’s disease

Authors	Compound	Year published	Receptors	Model	Result
Gołembiowska et al. (2013)	Istradefylline (KW-6002)	2013	A2A receptor antagonism	Rats treated with lipopolysaccharide (LPS)	Strengthening the therapeutic effect of L-DOPA
Zhu et al. (2014)	Istradefylline (KW-6002)	2014	A2A receptor antagonism	PD patients, a meta-analysis	Enhancement of the antiparkinsonian effects of levodopa; at a dose of 40 mg/d in supplementation a potential prospect for PD therapy
Sako et al. (2017)	Istradefylline (KW-6002)	2017	A2A receptor antagonism	PD patients, a meta-analysis	Phase 3 clinical trials: reduced time of “off” episodes for 40 mg/d dose, not effective as monotherapy
Gillespie et al. (2009)	Vipadenate (BIIB014)	2009	A2A receptor antagonism	Patients with PD	Not superior to placebo; development as an anti-Parkinsonian agent has been stopped
Brooks et al. (2010)	Vipadenate (BIIB014)	2010	A2A receptor antagonism	Healthy volunteers	A2A receptor occupancy is related to both dose and plasma levels of vipadenate (PET study)
Gyoneva et al. (2014)	Preladenate	2014	A2A receptor antagonism	Mice injected with 1-methyl-4-phenyl-1,2,3,6-tetrahydropyridine (MPTP)	Response of activated microglia to tissue damage, enhancement of the therapeutic effect of a low dose of L-DOPA
Hauser et al. (2014)	Preladenate	2014	A2A receptor antagonism	Patients with PD, two 12-week, phase 3, randomized, placebo-controlled, double-blind trials	2b phase the decrease in the “off” time was 1.0 h for the 5-mg dose and 1.2 h for the 10-mg dose, phase 3 kinetic trials: did not exceed placebo
Stocchi et al. (2017)	Preladenate	2017	A2A receptor antagonism	A randomized, 26-week, placebo- and actively-controlled, parallel-group, multicenter, double-blind study in adults diagnosed with PD for <5 years who were not yet receiving l-dopa or dopamine agonists	Preladenant is not effective as monotherapy at the studied doses (2, 5, 10 mg); for patients with early PD
Hauser et al. (2015)	Tosadenate (SYN115)	2015	A2A receptor antagonism	Patients with PD; phase 3, international, multicenter, randomized, double-blind, placebo-controlled, parallel-group, 3-group safety and efficacy study (part A) with an open phase (part B)	2b the reduction in “off” time was 1.2 h/d for doses of 120 and 240 mg. Phase 3 clinical trials: hematologic toxicity, agranulocytosis (in each group, a case of fatal septic shock).
Gołembiowska et al. (2013)	Caffeine	2013	A2A receptor antagonism	Rats treated with lipopolysaccharide (LPS)	Improvement of indicators of physical activity
Fuzzati-Armentero et al. (2015)	8-Ethoxy-9-ethyladenine (ANR 94)	2015	A2A receptor antagonism	6-OHDA-injected rats (male Sprague Dawley rats)	The combination of low doses of ANR 94 and metabotropic glutamate 5 (mGluR5) receptor antagonists provides greater therapeutic benefits than alone and also eliminates L-DOPA-induced dyskinesia
Ferreira et al. (2017)	SCH 58261 and ZM 241385	2017	A2A receptor antagonism	A2A receptor knock-out mice, SHSY5Y-derived neuronal cells	Prevents neuronal death; does not interfere with the oligomerization of α-synuclein (α-Syn), but reduces the percentage of cells expressing it
Gan et al. (2015)	NF449	2015	P2X1 receptor antagonism	H4 cells	Termination of α-synuclein aggregation
Marcellino et al. (2010)	A-438079	2010	P2X7 receptor antagonism	Rats with 6-OHDA lesion	Prevented 6-OHDA-induced depletion of striatum DA stores significantly but not completely
Carmo et al. (2014)	Brilliant Blue G (BBG)	2014	P2X7 receptor antagonism	Rats with 6-OHDA lesion	BBG (100 nm) prevented 6-OHDA-induced synaptosomal dysfunction
Wang et al. (2017)	Brilliant Blue G (BBG)	2017	P2X7 receptor antagonism	Intranigral lipopolysaccharide (LPS) rat model	Decreased microglial activation and preservation of DA neurons of the substantia nigra through inhibition of mitogen-activated protein kinase (p38 MAPK)
Wilkaniec et al. (2017)	Pyridoxal 5′-phosphate-6-azo-phenyl-2,4-disulfonate (PPADS)	2017	P2X7 receptor antagonism	SH-SY5Y-derived neuronal cells	Preventing abnormal calcium influx induced by α-synuclein
Qian et al. (2018)	MRS2578	2017	P2Y6 receptor antagonism	SH-SY5Y-derived neuronal cells	Delayed loss of neurons
Karoutzou et al. (2018)	3-amino-N ′- (2-chlorophenyl) adamantane-1-carbohydrazide (9g)	2018	NMDA and P2X7 receptor antagonism	HEK293 cells stably expressing the P2X7 receptor and primary culture granular neurons of rat cerebellum with NMDA receptors	The first double antagonist both receptors; inhibits the excessive activity of receptors in the low micromolar range while maintaining their physiological function

## Discussion: Perspectives and Challenges

The undoubted interest in P2X7R as a potential target for therapeutic agents is associated with certain successes in the treatment of inflammatory processes. However, the dual-functional role of P2X7, which is involved in both cell proliferation and apoptotic cell death shown in some studies, requires further study to understand the mechanisms involved in intracellular signal transmission with the participation of this receptor and imposes some restrictions on the use of these blockers in the development of neurodegenerative diseases. In addition, the presence of multiple isoforms of P2X7R in humans and experimental animals requires a meticulous approach when using certain receptor antagonists, since they are not always effective in some polymorphic types of receptors. An important aspect to consider is a detailed study of the mechanisms of the inflammatory response, since the nonselectivity of the antagonists chosen could lead to an imbalance in immune homeostasis.

A promising future direction is the search for therapeutic targets to provide microglial-cell-mediated neuroprotection. In this context, the therapeutic targets are adenosine receptors which provide a mix of phenotypes from proinflammatory (M1) to neuroprotective (M2). This approach to maintaining the balance of M1/M2 can ensure the survival of neurons in conditions of chronic inflammation.

Astroglia may also provide another course for further research. In astroglia, misfolded oligomeric proteins can result in a dysfunctional phenotype. In particular, α-synuclein is transferred from neurons to astrocytes during the development of neurodegenerative diseases. As a result, as mitochondrial dysfunction develops, the process of mitophagy is disrupted, and ER stress proceeds, which exacerbates neurodegenerative processes. Therefore, one of the tactics in maintaining the viability of neurons in conditions of neurodegeneration is the preservation of astrocytic function, which will be key for the survival of neurons. Further studies of the mechanisms of involvement of astrocytes in the development of neurodegenerative diseases will be important in the development of new therapeutic approaches.

## Conclusion

The purinergic signaling system is directly involved in the regulation of intracellular redox homeostasis and triggers a cascade of reactions that produce various forms of reactive oxygen and nitrogen. Excessive accumulation of ROS against the background of dysregulation of the antioxidant system leads to the oxidation of intracellular proteins, DNA, and lipids, which ultimately leads to disruption of mitochondrial energy. In modern research, extensive work is currently being devoted to describing the potential role of purinergic signaling in the development of various neurodegenerative diseases (Alzheimer’s, Parkinson’s, and Huntington’s diseases, multiple and amyotrophic lateral sclerosis). A common characteristic of these diseases is neuronal degeneration, which occurs against the background of neuroinflammation with sequential neurodegeneration because of the activation of P2X7Rs in microglia. In addition, the P2X7 receptor has specific binding sites for extracellular misfolded proteins that accompany the development of Alzheimer’s and Parkinson’s diseases and can spread between neurons and astrocytes by a prion-like mechanism.

**Figure 3.**
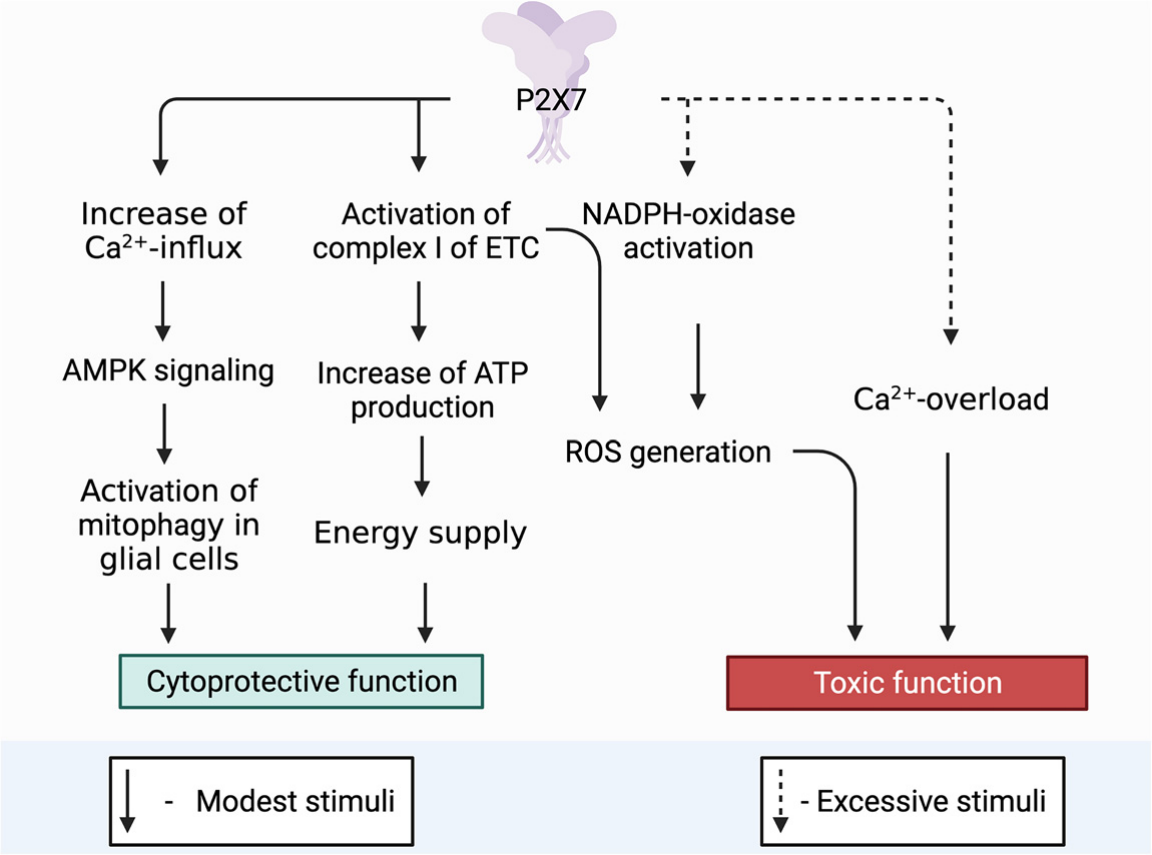
Role of P2X7 receptor in the functional activity of neurons. Toxic function: P2X7 triggers an intracellular signaling pathway for the activation of free radical oxidation processes and the accumulation of ROS, as a result, mitochondria are actively involved in the process of ROS production. ROS via AMPK signaling pathway can trigger mitophagy, thereby controlling the presence of defective mitochondria in the neuron removing them and ensuring cell viability. However, with excessive accumulation of ROS and a decrease in the activity of the antioxidant systems of the cell, the correct functioning of the PARKIN protein is blocked, which leads to inhibition of the mitophagy process. Misfolding of proteins, defective mitochondria accumulate in the cell. One of the defective proteins is α-synuclein that in turn has binding sites for the P2X7 receptor stimulating it and thereby increasing the Ca^2+^ concentration in the cell. An elevated level of Ca^2+^ blocks AMPK activity, after which the death of neurons can follow the path of necrosis. Cytoprotective function: P2X7 triggers mitophagy throught the Ca^2+^ increases which activation AMPK signaling pathway. It eliminates defective mitochondria thereby contributing to the survival of neuroglia and maintaining its viability in conditions of short-term stimulation. P2X7, localized on the outer mitochondrial membrane, captures the concentration of ATP in the cell and, with a decrease in its production, enhances the work of Complex I (NADH-DH) of the electron transport chain, as a result of which oxidative phosphorylation is stimulated and ATP production in the cell increases. *Figure Contributions*: Alexei V. Deykin described the cytoprotective function of P2X7 through activating AMPK-signaling pathway. Vladislav O. Soldatov performed the cytoprotective function by activation Complex I of ETC. Marina Y. Skorkina described the toxic function through ROS. Plamena R. Angelova developed the toxic function through Ca^2+^ overload.

The purinergic signaling system is the central trigger element in the development of mitochondrial dysfunction that accompanies neurodegenerative diseases. Among the whole family of purinergic receptors, P2RX7 is of decisive importance in future studies in terms of studying the involvement of this process in trophic, cytoprotective, and neuroinflammatory aspects (Fig. 3). Now, under what conditions one or another signaling path turned on is not clear, or what mutual signaling paths can switch one or another signal transmission path. It is necessary to find out what common substrates can be involved in the implementation of the opposite functions of this receptor.

Based on our generalizations, the primary role of P2X7 localized on neurons and glia in the formation of an inflammatory focus during prolonged uncontrolled stimulation is obvious. Under these conditions, P2X7 triggers an intracellular signaling pathway for the activation of free radical oxidation processes and the accumulation of ROS; as a result, mitochondria are actively involved in the process of ROS production. ROS via AMPK signaling pathway can trigger mitophagy, thereby controlling the presence of defective mitochondria in the neuron removing them and ensuring cell viability. However, with excessive accumulation of ROS and a decrease in the activity of the antioxidant systems of the cell, the correct functioning of the PARKIN protein is blocked, which leads to inhibition of the mitophagy process. In case of misfolding of proteins, defective mitochondria accumulate in the cell. One of the defective proteins is α-synuclein that in turn has binding sites for the P2X7 receptor stimulating it and thereby increasing the Ca^2+^ concentration in the cell. An elevated level of Ca^2+^ blocks AMPK activity, after which the death of neurons can follow the path of necrosis.

The cytoprotective function of P2X7 for glial cells has been described. With short-term stimulation of P2X7, the concentration of Ca^2+^ increases and activating AMPK signaling pathway. It triggers mitophagy and eliminates defective mitochondria thereby contributing to the survival of neuroglia and maintaining its viability.

The trophic function of the P2X7 receptor localized on intracellular membranes is an interesting and little studied aspect. In particular, P2X7, localized on the outer mitochondrial membrane, captures the concentration of ATP in the cell and, with a decrease in its production, enhances the work of Complex I (NADH-DH) of the electron transport chain, as a result of which oxidative phosphorylation is stimulated and ATP production in the cell increases. Thus, this receptor performs a trophic and sensory function by controlling the level of ATP inside the cell. In this regard, especially promising in terms of studying the mechanisms of development of neurodegenerative diseases is the search for signaling mediators involved in interactions between the P2X7 purinoreceptor localized on the neuron plasma membrane and on the membranes of intracellular organelles, such as mitochondria, phagosomes, and the nuclear membrane. Now is not clear the interaction between these receptors and through which signaling mediators they provide coordination in signal transmission.

Future research should focus on finding a set of signaling mediators that cause the P2X7 receptor to use different signaling pathways depending on the microenvironment and the concentration of signaling ligands. It is necessary to understand under what conditions the inflammatory role, trophic or cytoprotective function of the receptor, is realized, as well as how this receptor can be involved in the formation of the mitochondrial network and the fusion of intracellular membranes.

Purinergic receptors have multifaceted properties in the CNS: they not only promote neurotransmission and neuromodulation but also promote chemotaxis. Investigation of the role of the purinergic signaling system and understanding of the intracellular signaling cascades triggered by this system in neuropathological conditions allows researchers to focus their attention on the search for pharmacological targets that would minimize the harmful effects of pathologies and significantly improve the quality of life of patients. Therapeutic strategies in the treatment of neurodegenerative disease are aimed at suppressing neuroinflammation, organized with the participation of microglial cells and the P2RX7 receptor.

## Synthesis

Reviewing Editor: Francisca Bronfman, Universidad Andres Bello Facultad de Medicina Decisions are customarily a result of the Reviewing Editor and the peer reviewers coming together and discussing their recommendations until a consensus is reached. When revisions are invited, a fact-based synthesis statement explaining their decision and outlining what is needed to prepare a revision will be listed below. The following reviewer(s) agreed to reveal their identity: Evandro Fang, Nadia Rivero-Segura.

The current manuscript is an interesting review that highlights the opportunity areas in the field.

The review ‘P2X7 receptor and purinergic signaling: orchestrating mitochondrial dysfunction in neurodegenerative diseases’ summarized recent progress on the molecular pathways of the purinergic signaling, linking compromised purinergic signaling pathways to common neurodegenerative disease. This is a timely reivew summarized an important pathway in neuroscience.

Still there are some major and minor concerns that should be addressed before publication to increase the quality of the final manuscript.

Major concerns

1. Evidence of impaired purinergic signaling and P2X7 receptor in AD. Evidence provided in this review related to the link between P2X2 receptor and purinergic signaling in neurodegenerative diseases is not strong enough. It could be informative if the author(s) could make a table to summarize evidence of impaired P2X2 receptor and purinergic signaling across different neurodegenerative diseases, for example, AD, PD, ALS, HTT, etc.

2. Mitophagy, mitochondrial homeostasis, and neuroprotection. As mitochondrial dysfunction is a major topic in this review, it is necessary to include some key recent progress in the discovery of defective mitophagy as a driver of neurodegeneration via accumulation of damaged mitochondria (PMID: 30742114; PMID: 34992270).

3. Fig. 1. Fix/include the following points

a-To have ‘Ca2+’ in superscript;

b-Mitochondria are also impaired in astrocytes and microglia in common neurodegenerative diseases. Such changes should be reflected in Fig. 1

c-While apoptosis is a pathway for neuronal death, studies in human brain samples and animal models suggest that ‘necroptosis’, rather than ‘apoptosis’ is the major driver of neuronal death in Alzheimer’s disease (PMID: 28758999; PMID: 34646380). Please include these references and comments.

d-Also, the role of mitophagy in neuroprotection, as well as the contribution of compromised mitophagy in neurodegeneration should be shown

4. Include a figure that summarizes the role of the purinergic receptor related to mitochondrial function/dysfunction.

5. Provide a better image for the visual abstract

Minor concerns

1. Adding a figure showing the biochemical and crystal/structural information of the P2X7 receptor would help. Also, it would be informative to add the two main purines, ATP and adenosine, in the figures since they act as excitatory and inhibitory neurotransmitters, respectively.

2. Discuss necroptosis as a possible cell death mechanism when discussing in the following statement “We found the dual-functional role of purinoreceptor (P2X7) which is involved in both cell proliferation and apoptotic cell death”.

3. For the statement “Even though there are a number of known causative factors, mitochondrial dysfunction serves as a principal driver of neurodegenerative disorders (Abramov et al., 2017; Sukhorukov et al., 2020.; Kaur et al., 2021; Onyango et al., 2021)", more representative papers in the field should be included (PMID: 19081372; PMID: 25611507; PMID: 31577933).

4. It will be valuable for the manuscript to discuss future directions related to purinergic signaling, mitochondrial dysfunction, and neurogenerative diseases to highlight the relevance to continue the research in this area.
